# Inflammatory Bowel Diseases and Food Additives: To Add Fuel on the Flames!

**DOI:** 10.3390/nu11051111

**Published:** 2019-05-18

**Authors:** Rachel Marion-Letellier, Asma Amamou, Guillaume Savoye, Subrata Ghosh

**Affiliations:** 1 INSERM unit 1073, Normandie University, UNIROUEN, 22 boulevard Gambetta, F-76183 Rouen, France; asma.amamou@etu.univ-rouen.fr (A.A.); guillaume.savoye@chu-rouen.fr (G.S.); 2 Institute for Research and Innovation in Biomedicine (IRIB), Normandie University, UNIROUEN, F-76183 Rouen, France; 3 Department of Gastroenterology, Rouen University Hospital, 1 rue de Germont, F-76031 Rouen, France; 4 Institute of Translational Medicine, University of Birmingham, Birmingham B15 2TT, UK; sughosh@ymail.com

**Keywords:** colitis, food additive, diet, emulsifiers, high salt diet, inflammatory bowel diseases

## Abstract

Inflammatory bowel diseases (IBDs) develop in genetically predisposed individuals in response to environmental factors. IBDs are concomitant conditions of industrialized societies, and diet is a potential culprit. Consumption of ultra-processed food has increased over the last decade in industrialized countries, and epidemiological studies have found associations between ultra-processed food consumption and chronic diseases. Further studies are now required to identify the potential culprit in ultra-processed food, such as a poor nutritional composition or the presence of food additives. In our review, we will focus on food additives, i.e., substances from packaging in contact with food, and compounds formed during production, processing, and storage. A literature search using PubMed from inception to January 2019 was performed to identify relevant studies on diet and/or food additive and their role in IBDs. Manuscripts published in English from basic science, epidemiological studies, or clinical trials were selected and reviewed. We found numerous experimental studies highlighting the key role of food additives in IBD exacerbation but epidemiological studies on food additives on IBD risk are still limited. As diet is a modifiable environmental risk factor, this may offer a scientific rationale for providing dietary advice for IBD patients.

## 1. Introduction

The most common types of inflammatory bowel diseases (IBDs) are Crohn’s disease (CD) and ulcerative colitis (UC). IBD etiology is unknown, but IBDs develop in genetically predisposed individuals in response to environmental factors and the result of an exacerbated mucosal immune response to intestinal microbiota. IBDs are concomitant conditions of industrialized societies [1]. Indeed, IBD prevalence has continued to increase in Western countries, and newly industrialized countries in Asia, the Middle East, Africa, and South America have exhibited a rapid increase of IBD prevalence [1].

Environmental factors are supposed to play a decisive role in the pathogenesis of IBDs. Diet is considered to be a potential culprit and we previously reviewed the potential effect of specific nutrients in IBDs [2]. With this study, we aimed to focus on another potential dietary culprit: food additives. 

Consumption of ultra-processed food (UPF) has increased over the last decade, in particular in industrialized societies [3,4,5], and studies from the French web-based NutriNet-Santé cohort have found an association between UPF consumption and chronic diseases, such as a higher cancer risk [6]. UPF now represent an important part of the diet of French individuals: UPF accounted for 16% of food consumed by weight, corresponding to 33% of total energy intake [7]. In addition, the authors also reported an association between these dietary patterns and a higher irritable bowel syndrome risk (OR Q4 vs. Q1 [95% CI]: 1.25 [1.12–1.39], *p*-trend < 0.0001) [7]. Similar associations between UPF consumption and chronic diseases are globally observed, such as metabolic syndrome in the US [4] or depression in Spain [5]. Further studies are now required to identify the potential culprit in UPF, such as a poor nutritional composition or the presence of food additives. In our review, we will focus on food additives, i.e., substances from packaging in contact with food, and compounds formed during production, processing, and storage.

## 2. IBD Patients and Dietary Beliefs 

Diet is a crucial point for IBD patients [8,9,10] and exclusion diets are commonly reported. In a French study, the dietary beliefs of 244 IBD patients were studied using a questionnaire of 14 items [8]. The majority of IBD patients (58%) believed that food can play a role in causing a relapse and this strongly affects quality of life because IBD patients may refuse outdoor dining for fear of causing IBD symptoms [8]. In a study conducted in the UK, 400 IBD patients received a dietary questionnaire and data from this study were in accordance with the French study. In this study, deteriorating symptoms were associated with certain foods from 60% of IBD patients [10]. Similar results were found in a Dutch study conducted in 294 IBD patients through an online questionnaire [9]. Interestingly, 81% of IBD patients from the Dutch study reported that their main nutritional knowledge came from their own experience, while 71% reported that they received professional dietary advice mainly from dieticians [9]. In a Spanish study, the majority of IBD patients (86%) refrained from eating some foods to avoid a worsening of flare-ups [11]. In pediatric IBD populations, food avoidance is also commonly reported for 53% of patients [12].

## 3. A Pinch of Salt

**Figure 1:**
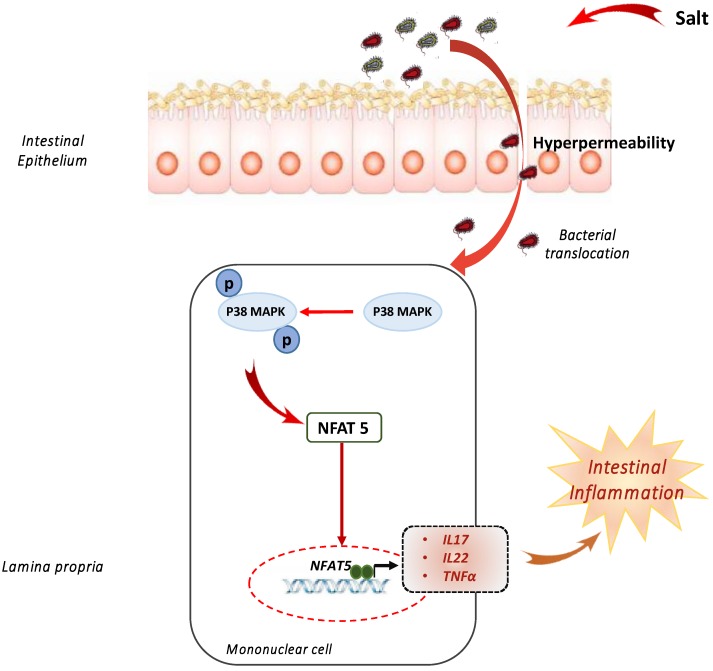
High-salt diet promotes intestinal inflammation. High-salt diet exposure led to higher intestinal permeability and dysbiosis. High-salt diet induced p38 MAPK phosphorylation and NFAT5 in lamina propria mononuclear cells activation with a subsequent expression of inflammatory cytokines expression such as IL17, IL22, and TNFα.

Processed food is a high provider of dietary sodium chloride (NaCl). Few recent studies have evaluated the potential impact of dietary salt in colitis models [13,14,15] (Figure 1). Tubbs et al. calculated the NaCl content of a series of available foods in grocery and fast food restaurants using the SELF Nutrition database, and they reported that these foods contained approximately 4% w/w NaCl [13]. Increased concentration of NaCl from 10 to 80 mM induced inflammatory cytokine production, such as the IL (interleukin)-23/IL-17 pathway in normal intestinal lamina propria mononuclear cells [14]. High-salt diet (HSD) exacerbated chemically induced colitis, while pharmacological inhibition of p38/MAPK abrogated its effect in both models [14]. Tubbs et al. demonstrated the deleterious effect of HSD in numerous colitis models, such IL-10^-/-^ or infectious colitis [13]. Interestingly, Aguiar et al. reported that dietary salt exacerbated colitis but by itself can trigger gut inflammation by increasing intestinal permeability and inflammatory histological score [15]. More recently, HSD also has been shown to have a deleterious impact on intestinal microbiota by decreasing *Lactobaccilus* levels and short-chain fatty acid production [16].

These studies raised the potential role of dietary salt as an environmental trigger for IBD development by creating a deleterious environment that is more vulnerable to inflammatory insults. 

Dietary phosphate has been less studied than dietary salt. Nevertheless, dietary inorganic phosphate is abundant in processed food as a food additive, in particular in fast foods and processed meats. Phosphate intake is two or three times higher [17]: 1655 mg/day for men and 1190 mg/day for women in the US compared to dietary reference intake at 700 mg/day in industrialized countries. Its deleterious effect on intestinal inflammation has also been demonstrated. Sugihara et al. fed Sprague–Dawley rats with a diet containing 0.5% to 1.5% phosphate for 7 days before colitis induction [18]. As standard animal diet contains approximatively 5000 mg/kg of phosphate, i.e., 0.5%, the range of phosphate from 0.5% to 1.5% used in the study mimics the human exposure range from 1- to 3-fold of the recommended dietary intake. Dietary phosphate exacerbates colitis by accentuating body weight loss by increasing disease activity index and by activating NF (Nuclear factor)-κB [18]. The authors of the study also found an in vitro increased inflammatory response by 2 mM phosphate in liposaccharide (LPS)-treated RAW264 cells [18]. 

To our knowledge, only one study has reported clinical data concerning salt consumption and IBDs [19]. In a US cohort from 194,711 women, the authors of this study reported that dietary intake of potassium (*P*_trend_ = 0.005) but not sodium (*P*_trend_ = 0.440) was inversely associated with risk of CD [19]. The authors did not observe any significant association between both dietary potassium and sodium and UC risk [19].

## 4. Take the Bitter with the Sweet

Carbohydrates are obviously part of a normal diet but their excessive consumption is a feature of the Westernized diet. In addition, industrialized food contains hidden sugars, such as lactose as a texturing agent in sausages. There is currently no evidence of an association between carbohydrate intake and IBD risk in epidemiological studies [20]. Results from the prospective EPIC (European Prospective Investigation into Cancer and Nutrition) study, when excluding cases occurring within the first 2 years after dietary assessment, found a positive association between a “high sugar and soft drinks” pattern and UC risk (1.68 [1.00–2.82]; *P*_trend_ < 0.05). When the authors considered the foods most associated with the pattern, high consumers of sugar and soft drinks were at higher UC risk only if they had low intakes of vegetables [21], but the amount of consumed sugar was not mentioned. Those with pediatric IBDs had a lower intake of carbohydrates compared to the general population, in particular a decreased intake of food types high in sugar [12].

Polysaccharides are commonly added in UPF as emulsifiers, coating agents, stabilizers, or bulking agents. These ingredients enable a better palatability and a longer shelf like of these products. Some of these ingredients have been studied in experimental models of IBDs [22,23,24,25]. 

Nickerson et al. focused their studies on the effect of the dietary polysaccharide maltodextrin to bacterial-induced intestinal inflammation [22,23,24]. Maltodextrin is commonly found in processed food and medications. Nickerson et al. demonstrated that maltodextrin can promote *Salmonella* survival in multiple cell types [24]. They also studied the effect of maltodextrin on CD-associated adherent-invasive *Escherichia coli* (AIEC) and found that the presence of maltodextrin enhanced AIEC adhesion by a better biofilm formation [22]. Maltodextrin consumption may promote intestinal dysbiosis and contribute to disease susceptibility. In a recent study, maltodextrin in drinking water exacerbated intestinal inflammation in an indomethacin-induced enteropathy model or in DSS (Dextran sodium sulfate)-colitis. The mechanisms behind maltodextrin’s deleterious effects involved endoplasmic reticulum stress and led to mucin-2 depletion [25].

The consumption of sweeteners is becoming more and more frequent in the US general population. A recent study on 16,942 participants reported that 25% of children and 41% of adults in the US consumed foods and beverages containing low-calorie sweeteners [26]. Two large Swedish prospective cohort studies consisting of 83,042 participants did not find any association between sweetened beverages consumption and risk of later-onset CD or UC [27]. In South Korea, food additive consumption (such as sucralose) cannot explain the increased incidence of IBDs [28]. Increased IBD incidence started before the 1980s in South Korea, while sucralose was only approved in 2000 [28]. A recent study demonstrated the deleterious impact of an artificial sweetener called Splenda in experimental IBDs [29]. Splenda contains 1% sucralose and 99% maltodextrin. The authors of this study investigated the impact of 6 weeks of supplementation with Splenda in SAMP1/YitFc mice that spontaneously developed ileitis. Splenda supplementation induced ileal myeloperoxidase activity and promoted dysbiosis, in particular an expansion of proteobacteria [29]. 

Carrageenan is a polysaccharide commonly used as a food additive. In experimental models, it is also commonly used to induce an inflammatory response in vitro or in vivo [30,31]. The daily consumption of carrageenan in the US diet is approximatively 250 mg per adult. Recently, a small clinical trial was conducted to evaluate the potential benefit of a carrageenan-free diet in UC patients [32]. Patients were instructed to follow a carrageenan-free diet by avoiding carrageenan-containing-food and received either 100 to 200 mg of carrageenan capsules or placebo capsules. Carrageenan-supplemented patients had some higher inflammatory parameters, such as a higher clinical disease index and higher IL-6 and fecal calprotectin [32]. Carrageenan also has an impact on intestinal microbiota [33]. Colitis induction by carrageenan at 20 mg/L in drinking water decreased the level of an anti-inflammatory bacteria *Akkermansia muciniphila* [30]. 

Chassaing et al. did a remarkable study on two emulsifiers, carboxymethylcellulose (CMC) and polysorbate-80 (P80), on gut barrier [34,35,36]. They added emulsifiers in the mouse drinking water at a relative low level for 12 weeks in wild-type or IL-10-deficient mice [35]. Dietary emulsifiers altered gut microbiota composition and induced hyperpermeability [35]. Emulsifiers promote colitis in IL-10^-/-^ mice by increasing inflammatory markers such as fecal lipocalin-2 or myeloperoxidase activity [35]. They demonstrated microbiota involvement in mediating the emulsifier effect by transferring intestinal microbiota from emulsifier-treated mice into unexposed germ-free mice; upon doing so, they observed that colonized germ-free mice had the same response [35]. They confirmed in a second study the direct impact of the emulsifiers on the microbiota using a mucosal simulator of the human intestinal microbial ecosystem; they observed that emulsifiers-promoted flagellin levels [36]. In another study, Chassaing et al. investigated whether emulsifiers can impact colon carcinogenesis [34]. They exposed mice with colitis-associated cancer to the same emulsifiers for 13 weeks and they observed that emulsifiers promote carcinogenesis through intestinal microbiota alteration, the establishment of a pro-inflammatory environment, and increased proliferation marker Ki67 [34]. Interestingly, they demonstrated in a recent study that emulsifiers also had a significant impact on anxiety [37], and psychological co-morbidities are commonly observed in IBD patients and IBD experimental models [38]. 

One-third of IBD patient underwent irritable bowel syndrome-like symptoms [39] and a recent meta-analysis confirmed that dietary restriction of fermentable oligosaccharide, disaccharide, monosaccharide, and polyol (FODMAP) reduced functional gastrointestinal symptoms such as bloating [40]. In heathy volunteers, mechanisms behind low-FODMAP diet involved decreased *Bifidobacterium* and reduced breath hydrogen [41].

## 5. Put the Pedal to the Metal

Aluminum is an abundant element commonly used in our environment. It is used in processed foods and cooking materials such as food packaging or foils. Cosmetic products and drugs are also a source of aluminum. The European Food Safety Authority set the tolerable weekly intake of 1 mg aluminum/kg body weight. Pineton de Chambrun et al. investigated the dietary effect of aluminum in colitis models. The strength of this study from Lille University is that the aluminum dose of 1.5 mg/kg/day that they used was relevant to human exposure [42]. Four weeks of oral administration of aluminum has been shown to worsen chemically-induced colitis models. Indeed, macroscopic and histologic scores and myeloperoxidase activity were higher in colitis mice exposed to aluminum. They also found a similar effect in IL-10^-/-^ mice. Exposure of epithelial cells lines with aluminum phosphate increased constitutive or LPS-induced cytokines mRNA level. Interestingly, aluminum exposure also delays mucosal repair capacity by inhibiting proliferating processes [43]. IBD patients often suffer from visceral pain even in remission periods [39], and a recent study from the same team has shown that dietary exposure of aluminum also induced visceral hypersensitivity in rodents [44]. This effect persisted over time after treatment cessation and involved mast cell activation [44].

## 6. To Add or Not to Add

Titanium dioxide (TiO_2_) is a food pigment which increases opacity and confers a white color [45]. Dietary sources of TiO_2_ are numerous, from sweets to toothpaste, and it is often used to provide a natural whiteness and opacity to foods, such as icing on cakes. TiO_2_ has an optimal particle diameter of 200–300 nm, but a nano-sized fraction can be found because of manufacturing processes. These TiO_2_ nanoparticles, in Europe listed as E171, are commonly used as a food additive. We previously investigated the effects of microparticles on macrophages from CD patients, and we observed that microparticles alone did not induce an immune response [46]. By contrast, microparticles act as adjuvants to induce potent cytokine responses in the presence of bacterial antigens such as LPS [46]. In addition, macrophage function has been shown to be impaired in the presence of high concentrations of microparticles [46]. Houdeau et al. investigated the specific effect of E171 on gut barrier [47] at a relevant range for human exposure, because modeling of human exposure to TiO_2_ estimates an exposure of 2–3 mg/kg/day for children under the age of ten [48]. After one week of dietary exposure to TiO_2_ at 10 mg/kg, titanium can be detected in Peyer’s patches in rats [47] and it is associated with an unfavorable immune cell balance: an increased CD11b/c^+^ CD103^+^ MHC-II^+^ dendritic cell proportion and a decreased proportion of regulatory T cells [47]. This TiO_2_ effect is not transient because it lasts after 100 days of TiO_2_ treatment [47]. In addition, long-term exposition for 100 days is associated with low-grade colon inflammation and induced aberrant crypt foci in a chemically induced carcinogenesis model [47]. The effect of dietary exposure to titanium dioxide at 50 to 500 mg/kg on colitis development has been investigated [49]. Dietary titanium dioxide exacerbates acute DSS colitis by inducing a shortening of the colon and increasing infiltration and histological inflammatory scores [49]. The same experiment was performed in inflammasome Nlrp3-/- mice, and the results showed that dietary TiO_2_ at 500 mg.kg^−1^ did not reproduce colitis exacerbation in Nlrp3/- compared to the response of the wild type mice. The mechanisms behind the dietary effect of TiO_2_ on colitis involved NLRP3. In vitro works demonstrated the accumulation of TiO_2_ in human intestinal epithelial and macrophage cell lines in a dose-dependent manner [49]. In addition, TiO_2_ at 20 to 100 µg/mL stimulates oxidative stress and increased intestinal permeability in human intestinal epithelial cell lines. Rogler et al. found that patients with active UC exhibited increased blood levels of titanium [49]. In extra-intestinal organs, dietary exposure at 2.5 to 10 mg/kg to TiO_2_ for 6 months upregulated renal TGFβ and SMAD, signaling pathways involved in fibrosis [50]. We speculate that dietary TiO_2_ may also promote IBD-induced intestinal fibrosis, but this effect has not been yet documented. Two recent studies have proposed a dietary intervention to counteract TiO_2_-induced inflammation in mice. The administration of both flavonoids naringenin or quercetin inhibits TiO_2_-induced arthritis by decreasing cytokine production and oxidative stress [51,52]. 

Oral bisphenol A is a chemical used in food packages. Oral bisphenol A at a dose lower to the tolerable daily intake was able to induce intestinal hyperpermeability in rats, and this effect occurs in a dose-dependent manner [53]. In trinitrobenzene sulfonic acid (TNBS)-induced colitis, perinatal exposure of dietary bisphenol A increased myeloperoxidase activity in female rats but not in male rats [53]. 

Dietary bisphenol A induced visceral hypersensitivity in response to colorectal distension [53]. A recent study highlighted the effect of bisphenol A in chemically induced colitis in ovariectomized female mice, and the authors demonstrated that bisphenol A exposure led to a worsened colitis through intestinal microbiota metabolites, such as a decreased level of tryptophan [54]. 

Food heat treatment leads to neoformed compounds such as Maillard reaction products. Exposure to these Maillard reaction products alleviated inflammatory response [55] and dysbiosis in colitis models [56]. 

## 7. Exclusion Diets 

Exclusion diets are commonly followed by IBD patients [57]. These exclusion diets have poor or no scientific rationale but expose IBD patients to nutritional deficiencies. The last European Society for Clinical Nutrition and Metabolism (ESPEN) guidelines about clinical nutrition in IBD patients declared that there is no IBD diet in active disease (strong consensus—96% agreement) [58]. We will limit our section to a few exclusion diets that may explain some benefits to limit food additive exposure. 

Exclusive enteral nutrition (EEN) is recommended as first intention treatment to induce remission in children or teenagers with CD [58], although EEN is not recommended in adult patients. The mechanisms behind EEN are not fully understood, but it may involve dysbiosis correction or gut barrier maintenance [59]. EEN mechanisms may also involve the absence of specific deleterious food components, such as food additive exposure. 

There is also concern about the presence of pesticides in the food chain, although there is still no evidence connecting pesticides to IBDs [60]. A recent study from the NutriNet French cohort found that more frequent organic food consumption decreased cancer risk [61].

We previously mentioned the effect of nanoparticles in gut barrier dysfunction in preclinical models, and two clinical trials have investigated the impact of low or microparticle-free diet in IBD patients. The first one was a very small trial in 20 CD patients that showed a beneficial effect of low or microparticle-free diet in ileal CD patients, such as decreased corticosteroids intake [62].

The second study included 84 active CD patients for 16 weeks [63] and the authors did not find any benefit of the microparticle exclusion on inflammatory disease activity index, fecal calprotectin, or intestinal permeability [63]. We previously reported the effects of carrageenan on gut barrier in preclinical models. A small pilot study investigated the effect of carrageenan-free diet in 12 UC patients [32]. Three out of ten UC patients who followed the carrageenan-free diet for 12 months relapsed compared to three out of five patients in the control group (RR (Relative risk) 0.50, 95% CI 0.15 to 1.64), but it may be difficult to conclude that there are potentially beneficial effects because of the very small size of the study. 

Lactose-free diets are common in IBD patients. In a small recent study on 78 IBD patients from Iceland, 60% (47 patients) reported limiting their dairy consumption or even excluding it, but only eight of them used calcium supplements [64]. Acquired lactase deficiency is commonly reported in CD patients, in particular in proximal CD. In a clinical trial in 77 UC patients published in 1965, milk-free diets had only a benefit for one out of five patients [65] and did not justify dairy-restricted diets in all IBD patients. In 29 pediatric UC patients, exclusion of cow milk proteins had no influence on induction and/or maintenance of remission [66]. 

Paleolithic diet is another exclusion diet without scientific rationale. Its principles are that our gastrointestinal system did not evolve alongside our modernized diet and may explain numerous inflammatory diseases. This diet excludes a lot of foodstuffs, such as ultra-processed foods or cereals, and is limited to wild meats and a non-cereal, plant-based diet. To our knowledge, the only published data about paleo diet and IBDs is a Hungarian case report about a teen CD patient’s refractory to treatments. This CD patient had a clinical remission after 2 weeks on a paleo diet and was still in remission 15 months later [67]. Extrapolation from a unique case control study is not permitted, and exclusion of so many foodstuffs may be associated with numerous nutritional deficiencies. 

One-third of IBD patients underwent IBS-like symptoms, and partial exclusion of some fermentable components may be associated with benefits to functional symptoms. For example, in a small study with 16 CD patients in remission, a strict exclusion diet of wheat and dairy products for 2 weeks significantly decreased function symptoms such as abdominal pain [68]. In a prospective study with 89 IBD patients in remission, a low-FODMAP diet for 6 weeks reduced IBS-like symptoms while it increased quality of life in patients [69]. 

Exclusion diets are not recommended and may expose patients to numerous nutritional deficiencies. In addition, cofounding factors are often observed, leading to unjustified dietary exclusion. Recently, a double-blind clinical trial identified that fructan composition, instead of gluten, induced functional symptoms in patients with self-reported non-celiac gluten sensitivity [69]. Similar results were previously reported concerning lactose intolerance [70]. Avoiding some foods has been identified as a risk factor for malnutrition in IBD patients in a Spanish cohort [71]. Of note, malnutrition increased further complications, such as higher surgery and hospitalization rates, and malnutrition is associated with altered quality of life in IBD patients [11,72]. In addition, exclusion diets may limit socializing and dining because many IBD patients reportedly refused dining out because doing so would not conform with their exclusion diet [11]. Exclusion of dairy products such as in a lactose-free diet or paleo diet may increase osteoporosis risk in IBD patients, while gluten-free diets are associated with decreased microbiota diversity. 

## 8. Conclusions

Diet is a modifiable environmental risk factor and food additives may act as potentiators of disease. Diet is a crucial point for IBD patients and exclusion diets appear to offer an alternative allowing for relative control of disease development. Nevertheless, the last ESPEN guidelines did not recommend any exclusion diet for induction or maintenance of remission in IBD patients but advised IBD patients to undergo counselling by a dietitian to avoid malnutrition. In addition, it has been recently reported that IBD patients in remission have an unbalanced dietary profile for essential nutrients [73]. Rather than exclusion diets, home-made food may be prioritized to decrease food additive amounts and enable patients to control their exposure to many hidden ingredients, such as added salt or sugars. Home-made food is already recommended in the last nutritional guidelines in France and, contrary to exclusion diets, cooking can represent a form of a convivial time, allowing a better quality of life with increased socialization. In addition, obesity is more and more frequent in IBD patients [74] and food preparation from scratch may be associated with a decreased risk of obesity [75] or decreased amount of dietary energy from ultra-processed food [76].

## Abbreviations

AIEC	Adherent-invasive *Escherichia coli*
CD	Crohn’s disease
CMC	carboxymethylcellulose
EEN	exclusive enteral nutrition
ESPEN	European Society for Clinical Nutrition and Metabolism
FODMAP	fermentable oligosaccharide, disaccharide monosaccharide, and polyol
HSD	High-salt diet
IBD	inflammatory bowel diseases
LPS	liposaccharide
NFAT5	nuclear factor of activated T cells 5
NLRP	NOD-like receptor pyrin
P80	polysorbate-80
SCFA	short-chain fatty acid
SMAD	small mothers against decapentaplegic
TNBS	trinitrobenzene sulfonic acid
TiO2	titanium dioxide
UC	ulcerative colitis
UPF	ultra-processed food
